# Navigating Diagnostic Uncertainty: A Case Report on Anesthetic Management of Mechanical Thrombectomy in a Patient With Occult Fibromuscular Dysplasia

**DOI:** 10.1155/cria/4688749

**Published:** 2026-08-30

**Authors:** Samuel Grant Volpe, Muskan Sidhu, Aariya Srinivasan, Sunpreet Tandon, Rohan Thakkar, Sabry Ayad

**Affiliations:** ^1^ Department of Anesthesiology Research, Cleveland Clinic Foundation, Cleveland, Ohio, USA, clevelandclinic.org; ^2^ Outcomes Research Consortium, Houston, Texas, USA, or.org; ^3^ Staff Radiologist, Cleveland Clinic, Fairview Hospital, Cleveland, Ohio, USA, clevelandclinic.org; ^4^ Staff Anesthesiologist, Cleveland Clinic, Fairview Hospital, Cleveland, Ohio, USA

## Abstract

Fibromuscular dysplasia (FMD) is a rare, nonatherosclerotic vascular disease that predisposes patients to arterial stenosis, dissection, and stroke, often with delayed diagnosis due to nonspecific symptoms. We present a 30‐year‐old female with a history of migraines and recent whiplash injury who developed acute ischemic stroke from right internal carotid artery occlusion. Following neurological deterioration, she underwent emergent mechanical thrombectomy under general anesthesia. Airway management incorporated manual in‐line stabilization to minimize cervical vessel stress, and hemodynamics were tightly controlled to balance cerebral perfusion and hemorrhagic risk. Postoperative imaging revealed arterial irregularities and a dissecting pseudoaneurysm consistent with previously undiagnosed FMD. The patient recovered with supportive care and was discharged on dual antiplatelet therapy. This case highlights the anesthetic challenges of managing acute stroke in the setting of occult vascular pathology and underscores the importance of maintaining suspicion for FMD in young patients with cerebrovascular events.

## 1. Introduction

Fibromuscular dysplasia (FMD) is a nonatherosclerotic, noninflammatory systemic vascular disease that can result in arterial stenosis, occlusion, aneurysm, or dissection. Despite extensive research, the etiology of FMD remains unclear. Only 5% of patients with the condition report an affected family member, suggesting environmental factors may play a larger role than genetics in its development. Vascular abnormalities from FMD arise most commonly in the renal arteries and extracranial carotid and vertebral arteries, but have been reported in nearly every arterial bed throughout the human body [1]. In the United States, FMD is considered a rare condition that primarily affects females below the age of 50, with an estimated overall prevalence of 12 cases per 100,000 individuals [2]. FMD is commonly diagnosed via incidental imaging findings, but in symptomatic patients, it classically presents with renovascular hypertension and stroke in young adults. Transient ischemic attacks and ischemic strokes manifest due to various mechanisms, including hypoperfusion, intracranial arterial stenosis, and emboli from a thrombosis in the area of stenosis or dilation [3].

Importantly, there are significant delays in diagnosis of FMD from the first reported onset of clinical symptoms, leading many patients to be diagnosed only after suffering a severe complication [4]. Many attribute this to the fact that FMD initially presents with a multitude of nonspecific symptoms such as headache, migraine, tinnitus, and hypertension. When complications such as stroke arise in these patients, it is considered a neurological emergency, and anesthesiologists play a critical role in maintaining hemodynamic stability, for which the benchmark for stability varies by factors such as occlusion location and stage of the procedure [5]. In addition, anesthesiologists must be prepared for airway management due to worsening neurological status throughout the procedure as well as tightly monitoring the anticoagulation status of the patient.

In this report, we present a case of a 30‐year‐old female with a history of migraines and a recent whiplash injury who underwent emergent mechanical thrombectomy for acute stroke due to occlusion of the internal carotid artery. Given the etiologic uncertainty of the stroke at the time of operation, we will use this report to discuss its unique anesthetic challenges, such as decisions to use general anesthesia (GA) over conscious sedation (CS), airway management considerations including the rationale for manual in‐line stabilization, the complexities of hemodynamic management, and postoperative implications, while highlighting the importance of certain intraoperative decisions in a patient with previously unrecognized systemic vascular disease. We further emphasize that in young patients presenting with cervical arteriopathy, an underlying nonatherosclerotic vasculopathy should be actively investigated rather than presumed absent, even when a recent trauma offers a fitting mechanistic explanation.

This case report was prepared and is reported in accordance with the CARE (CAse REport) guidelines. A completed CARE checklist and a structured, chronological patient timeline are provided (Table A1: CARE checklist; Table A2: Patient timeline).

### 1.1. Case History, Examination, and Diagnosis

A 30‐year‐old female patient presented to the emergency department with complaints of headache, slurred speech, and right‐sided weakness. Two weeks earlier, she had sustained a whiplash injury at a concert. Her past medical history was notable for bipolar disorder, attention‐deficit disorder, and migraine headaches without aura. Her past family history was notable for a stroke in her mother around a similar age to the patient. Additional history relevant to young‐adult stroke was reviewed, which revealed norethindrone contraceptive use, no history of smoking or substance use, and nulliparity. She reported experiencing a right‐sided headache the night before presentation and awoke with slurred speech and right‐sided facial numbness and weakness in the right upper and lower extremities with an NIHSS score of 3.

**FIGURE 1:**
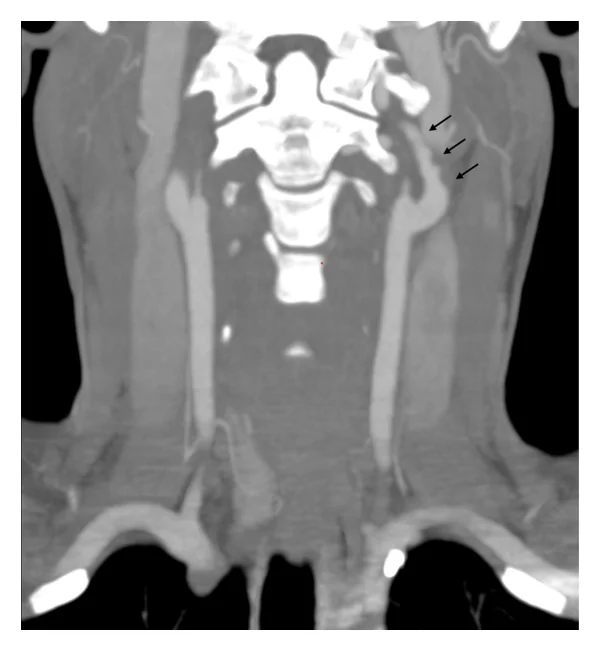
Coronal view. Left internal carotid artery showing fibromuscular dysplasia (FMD).

**FIGURE 2:**
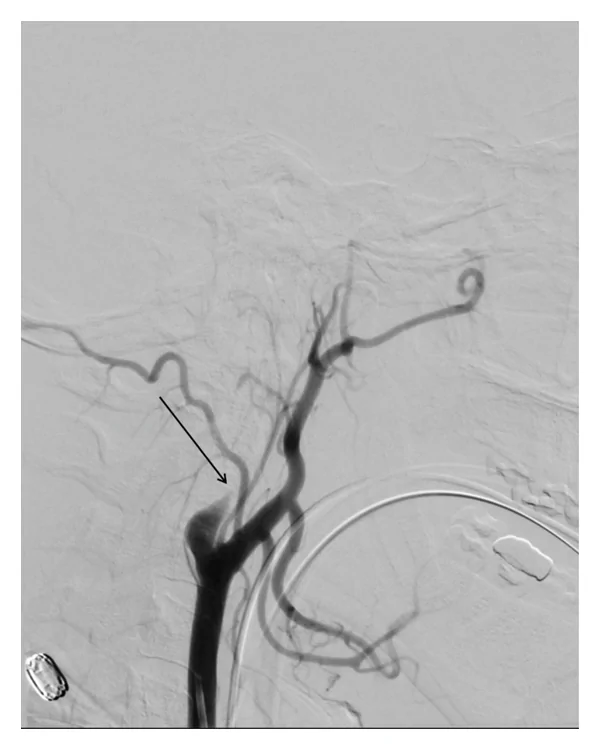
Angiogram showing right internal carotid artery occlusion.

**FIGURE 3:**
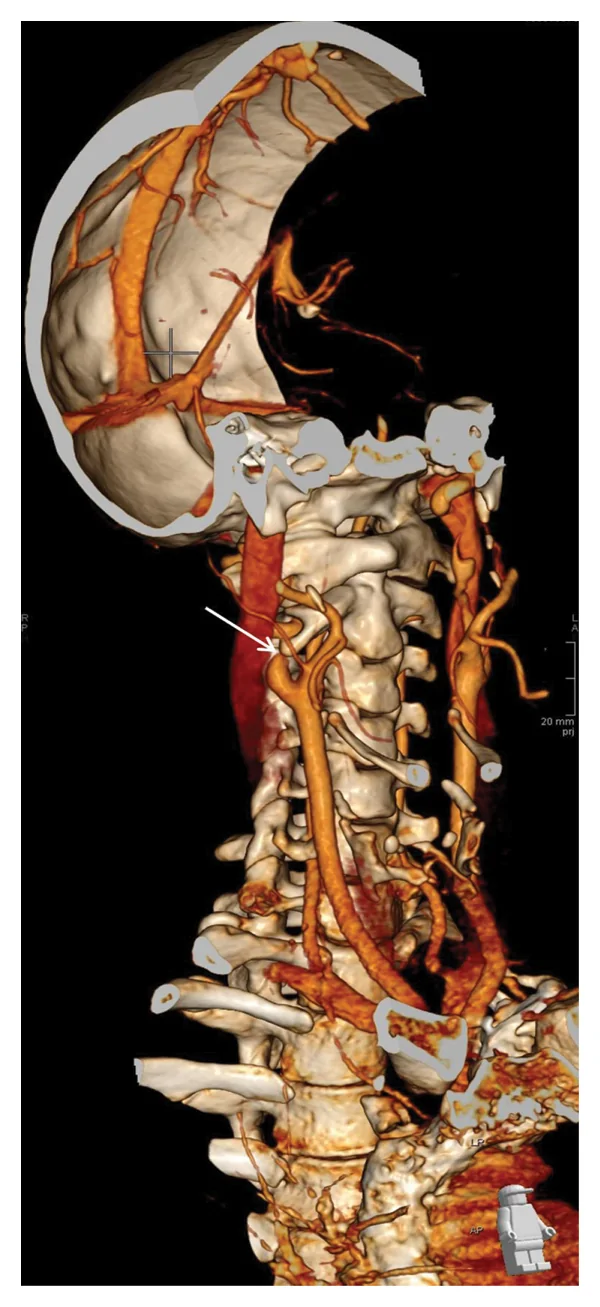
Right internal carotid artery occlusion. Oblique view 3D reconstruction.

**FIGURE 4:**
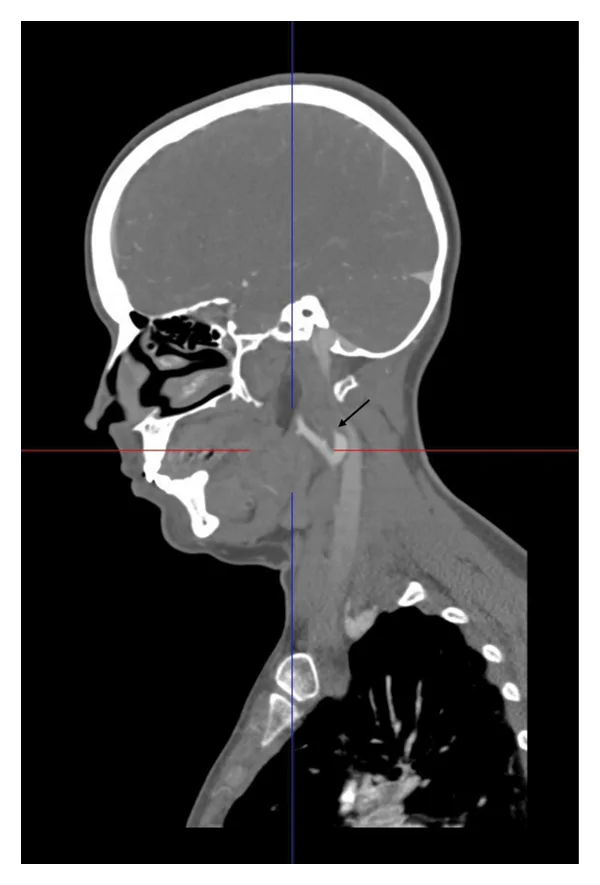
Right internal carotid artery occlusion. Sagittal view.

Alberta Stroke Program Early CT Score (ASPECTS) on the initial noncontrast CT was 10. CT perfusion was not obtained. Subsequent CT angiography (CTA) of the head and neck revealed occlusion of the right internal carotid artery (RICA), high‐grade stenosis of the left internal carotid artery (LICA), and mild narrowing of the distal cervical left vertebral artery consistent with arterial dissection (Figures 1, 2, 3, and 4).

**FIGURE 5:**
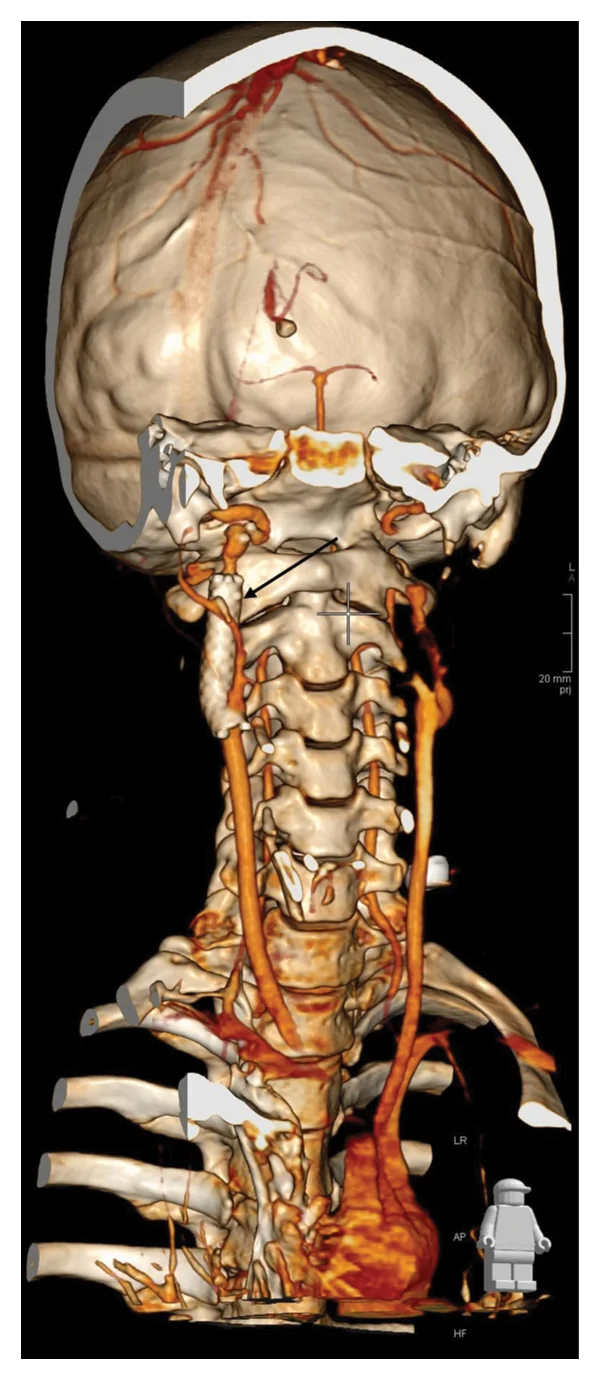
Postoperative 3D reconstruction imaging showing the stent placed.

As she was outside the therapeutic window for intervention, she was admitted for medical management and stroke care. Later that evening, a rapid response was initiated for new‐onset left‐sided weakness and facial droop. At this time, the bedside exam revealed a worsening NIHSS score of 11. Repeat CTA imaging demonstrated increased attenuation in the right middle cerebral artery (MCA) territory. Given the development of profound left‐sided deficits, neurointerventional radiology was consulted and recommended acute intervention. The patient subsequently underwent emergent thrombectomy and stent placement under GA (Figure 5).

### 1.2. Management

The patient was intubated using in‐line manual stabilization, and an arterial line was placed for continuous monitoring. Rapid sequence induction was achieved with 200 mg of propofol and 200 mg of succinylcholine followed by 50 mg of rocuronium. In addition, 30 mg of esmolol was administered to control blood pressure and to mitigate tachycardia and hypertension, which can increase the risk of hemorrhagic transformation after reperfusion. The patient’s blood pressure peaked at 178/115 approximately 7 min after the procedure began, likely secondary to intubation and procedural stimulation.

Following the rise, the patient’s blood pressure stabilized, and no additional beta‐blocking agents were required. Phenylephrine infusion was initiated at 50 mcg/min and then adjusted to 25 mcg/min in response to a mean arterial pressure (MAP) decrease to 74 mmHg. Dexamethasone (4 mg) was administered to mitigate postoperative nausea and vomiting (PONV), along with 1635 mcg of tirofiban as peri‐procedural antiplatelet therapy.

Additional agents included 10 mg of ephedrine, a single 200 mcg bolus of phenylephrine, and 0.1 mg of glycopyrrolate to manage reflex bradycardia related to phenylephrine administration. Reversal of neuromuscular blockade was achieved with 20 mg of sugammadex, and lactated Ringer’s solution was provided for fluid management. After the surgery, the patient emerged from anesthesia, was extubated, and transferred to the surgical intensive care unit for ongoing neurological surveillance and hemodynamic monitoring.

### 1.3. Outcomes and Follow‐Up

The patient was monitored in the neurocritical ICU for 2 days and was transferred to the regular nursing floor on Hospital day 3. Given her family history and recent vascular surgery for stenoses, a CTA was obtained with brain‐to‐pelvis coverage. Notable findings included dilation of the left gonadal vein measuring up to 9 mm in the mid segment, 15 mm in the proximal segment, and severe compression of the left renal vein between the superior mesenteric artery and the aorta. A CTA of the neck with IV contrast revealed a new irregularity of the distal right cervical ICA with an anteriorly projecting outpouching measuring up to 7 mm, compatible with a dissecting pseudoaneurysm, as well as a new progressive area of mid left ICA stenosis.

After weighing the risks and benefits, the neurovascular team elected not to pursue further intervention. The patient was discharged on Hospital day 6 with a modified Rankin Scale (mRS) score of 3, on aspirin and clopidogrel for 3 months, and instructions to follow dissection precautions during this time. She was also advised to avoid air travel and to follow up with genetics and the FMD clinic. Six months after discharge, bilateral carotid artery ultrasound was performed, which revealed a tortuous vessel at the distal right ICA and an aneurysm of 8 mm. In addition, the patient was seen by genetics, where a comprehensive aortopathy genetic panel was performed, and no disease‐causing variants were identified. Given the lack of ability to point to a specific genetic cause, the patient’s history of dissection and stroke, and new vascular abnormalities, the patient was given a primary diagnosis of FMD.

## 2. Discussion

Although cerebrovascular events such as transient ischemic attacks, ischemic stroke, or cervical artery dissection are common presentations of FMD, the rarity of the condition makes it difficult to accurately diagnose FMD as the underlying etiology. In the case of this patient, with acute deteriorations in neurological status, mechanical thrombectomy under GA was undertaken prior to the FMD diagnosis. This case is representative of population trends, with diagnosis confirmation occurring after incidental imaging findings or life‐threatening vascular events. Despite the rarity of FMD as an underlying etiology, it is important for anesthesia providers to consider vascular pathologies while constructing a management plan that prioritizes GA, intubation techniques that minimize exacerbation of potential dissection, and meticulous control of hemodynamics.

This patient’s complex history of present illness includes a long‐standing history of migraines, a recent whiplash injury, initial presentation of worsening headache and right‐sided focal deficits, and acute decompensation later that day with left‐sided stroke symptoms. The temporal and shifting nature of these symptoms is best explained not by a single lesion but by the sequential involvement of both cerebral hemispheres in the setting of bilateral cervical arterial disease. Her initial right‐sided deficits, accompanied by dysarthria, localize to the left hemisphere and are most consistent with hypoperfusion in the territory of the high‐grade left ICA stenosis. The right ICA occlusion, although present on initial imaging, was likely compensated by collateral circulation at that time. Over the following hours, the right hemisphere decompensated, with the right MCA territory changes seen on repeat imaging producing her left‐sided deficits and prompting emergent intervention.

Deciding whether this presentation is more likely due to traumatic injury or due to an underlying vasculopathy has direct consequences for the anesthesiologist, as the suspected etiology shapes decisions about hemodynamic targets, airway technique, and the tolerance for neck manipulation, making a deliberate, structured differential essential. This need for breadth is reinforced by the etiologic profile of stroke in young adults (< 50 years of age), in whom a range of uncommon causes, both embolic and vascular, is disproportionately represented compared to older patients [6].

Accordingly, the differential diagnosis in a 30‐year‐old woman with multivessel cervical disease and a positive family history extends well beyond FMD with cervical artery dissection versus benign posttraumatic headache. It should include spontaneous cervical artery dissection, heritable connective tissue disorders, primary central nervous system vasculitis, reversible cerebral vasoconstriction syndrome (RCVS), substance‐related vasculopathy, and hypercoagulable states. In this patient, the basis for lowering suspicion for these additional entities included a negative urine toxicology, PT and APTT within normal limits, negative anticardiolipin antibody titers, Factor VIII and fibrinogen within normal limits, normal activated protein C resistance ratio, negative past medical history for secondary causes of CNS vasculitis (lupus, rheumatoid arthritis, hepatitis C, Behcet’s disease, and granulomatosis with polyangiitis), and negative family history of heritable connective tissue disorders.

Given that mechanical thrombectomy is the standard of care for eligible patients with acute ischemic stroke, an important peri‐procedural decision is the choice between GA or CS. For this patient, GA was selected. GA involves drug‐induced loss of consciousness, whereas CS produces a depressed level of consciousness in which patients can still respond purposefully to commands and tactile stimulation. From a peri‐procedural risk standpoint, GA has been associated with a higher incidence of intraprocedural hypotension and greater blood pressure variability compared to CS, while rates of postprocedural pneumonia appear similar between the two approaches [7, 8].

In the context of acute ischemic stroke, evidence regarding the optimal anesthetic approach has been mixed. A 2019 meta‐analysis demonstrated that protocol‐based GA was associated with reduced functional disability at 3 months; however, this was a single‐center study, and functional disability was the only outcome measured [7]. More recently, a randomized controlled trial published in 2025 found that patients undergoing endovascular therapy under GA with propofol and/or etomidate with succinylcholine had improved rates of 90‐day functional outcomes and higher rates of successful reperfusion compared to those treated with moderate sedation using fentanyl, midazolam, dexmedetomidine, and/or low‐dose propofol [9]. In contrast, a separate 2025 cohort study reported comparable recanalization rates between GA and CS in patients with low NIHSS anterior circulation strokes, though those managed with CS demonstrated better functional outcomes [10].

Given this patient’s severe stroke, its location, the uncertain underlying etiology, and the presence of a recent whiplash injury, GA was felt to be the most appropriate choice. Overall, the decision to use GA versus CS should be individualized, considering the stroke severity, patient‐specific risk factors, and the anticipated ability to tolerate the procedure.

Airway management is a key component of anesthetic planning for FMD patients to ensure that vascular pathologies are not exacerbated through improper technique. The primary vasculature at risk during intubation and extubation is the internal carotid arteries and vertebral arteries [11]. The rationale for manual in‐line stabilization is to minimize movements associated with positioning of the neck, such as flexion and extension, which can place stress on compromised vessels [12]. Video laryngoscopy is also preferred over direct laryngoscopy for a similar reason. Video laryngoscopy requires less neck manipulation for successful intubation and can help prevent precipitating or worsening an artery dissection. In addition, video laryngoscopy has higher success upon first attempt at tracheal intubation [13]. Minimizing the number of attempts required for successful intubation can also decrease the hemodynamic swings a patient might experience during the process.

Other options for airway management include supraglottic devices such as laryngeal mask airways (LMAs) or fiberoptic intubation. LMAs are generally considered suboptimal due to their limited control of ventilation, which can decrease cerebral perfusion in the setting of acute stroke. Fiberoptic intubation uses flexible bronchoscopes to achieve intubation and can be performed with the patient either awake or sedated. The primary rationale for using fiberoptic intubation is for complicated airway management, to provide better and more direct visualization of the anatomy. Patients are often pretreated with anxiolytics, which help modulate unnecessary movement of the neck anatomy. With the excellent visualization provided by fiberoptic intubation, it can remain a viable option for patients with FMD, alongside video laryngoscopy.

Hemodynamic management in the setting of arterial stenosis, and more specifically ICA occlusion, focuses on preserving cerebral perfusion by carefully balancing blood pressure. Although the exact optimal blood pressure parameters during and after procedure are unknown and remain a topic of research, the 2026 AHA/American Stroke Association Guideline for the Early Management of Patients with Acute Ischemic Stroke recommends maintaining blood pressure ≤ 185/110 mmHg prior to reperfusion therapy and < 180/105 mmHg following intravenous thrombolysis and/or endovascular therapy, while cautioning that intensive blood‐pressure lowering after reperfusion may be harmful [14]. Thus, while excessive hypertension increases the likelihood of hyperperfusion injury and intracranial hemorrhage, hypotension must also be avoided due to the risk of cerebral ischemia. Accordingly, most anesthetic strategies target a MAP at or slightly above the patient’s baseline, with strict avoidance of pressures below cerebral autoregulatory thresholds and use of cerebral oxygenation monitoring when available. More specifically, analysis of individual patient data from randomized controlled trials has shown that for patients undergoing endovascular therapy for acute ischemic stroke, a MAP < 70 mmHg for a cumulative time of more than 10 min, or a single sustained episode of 20 min below this threshold, was associated with poor outcomes [15].

In our patient, vasopressors such as phenylephrine were used to correct anesthesia‐induced hypotension and support cerebral perfusion in vulnerable territories distal to the occlusion, with the additional goal of optimizing flow through the compromised ICA. The decision to use phenylephrine was based on its practical availability as well as physiological rationale, as prior studies have shown that phenylephrine partly restores ICA flow reduced by anesthesia‐related hypotension [16]. Episodes of tachycardia or acute blood pressure surges are typically controlled with short‐acting beta‐blockers to limit myocardial oxygen demand and dampen abrupt hypertensive responses without causing prolonged hypotension. In our patient, esmolol was selected for these purposes. Anticholinergic agents also play a vital role in counteracting bradycardia and excessive vagal tone, thereby helping maintain adequate heart rate and cardiac output in the setting of increased afterload from vasopressor therapy.

Although there are no specific protocols for administering intraoperative anticoagulation during thrombectomy, when ICA occlusion is managed with endovascular stent–related interventions, peri‐procedural antiplatelet therapy is commonly employed. Agents such as tirofiban, a glycoprotein IIb/IIIa inhibitor, are used to reduce thromboembolic risk in high‐risk neurovascular procedures. With respect to corticosteroids, dexamethasone 4 mg was administered specifically for prophylaxis of PONV.

Given this patient’s age, family history, comorbid migraine, and multivessel involvement, the shared genetic and pathophysiological mechanisms linking migraine, FMD, and cervical artery dissection merit consideration. A genomewide investigation identified shared genetic architecture between FMD and common cardiovascular traits including migraine, intracranial aneurysm, and blood pressure, implicating loci such as PHACTR1 [17]. Consistent with this, extensive cervical‐to‐intracranial dissection has been reported in PHACTR1‐associated FMD in a young patient with phenotypic overlap to the present case [18]. These links reinforce the value of longitudinal genetics and FMD‐clinic follow‐up in this patient.

Since an FMD diagnosis was eventually established, the patient’s future anesthetic care should be properly planned to mitigate any adverse events. For patients with FMD, the main priorities of the anesthesia team should be to minimize further trauma to the area of vascular damage and to control hemodynamic swings.

International consensus recommends that all patients diagnosed with FMD, regardless of the initial site of vascular bed involvement, should subsequently undergo brain‐to‐pelvis angiographic imaging at least one time, especially prior to any future procedures requiring anesthesia [19]. This patient’s postoperative imaging revealed not only the left proximal ICA dissecting pseudoaneurysm but also renal vascular involvement, which holds significance for managing her hemodynamics. With renal artery stenosis present, there is potential for baseline renovascular hypertension, which must be controlled perioperatively. Additional arterial bed involvement can also lead to different prioritization of hemodynamic parameters to ensure that dissections are not propagated, while adequate perfusion is maintained.

The decision in this case to use manual in‐line stabilization and video laryngoscopy is appropriate for further procedures to appropriately manage hemodynamic swings during intubation and minimize neck manipulation. The choice between GA and CS remains individualized based on trauma history and procedure type, and CS should be seriously considered in future procedures due to its lower incidence of intraprocedural hypotension and blood pressure variability. Overall, examining the decisions involved in this patient’s care retrospectively reveals the importance of consistently monitoring the progression of FMD. Imaging is possible before elective procedures, but in case of emergent situations requiring surgical interventions, it is important to have up‐to‐date information to make safe and well‐informed anesthesia decisions.

## 3. Conclusion

This case highlights FMD as an initially unrecognized but consequential case of acute‐large vessel stroke in a young patient and underscores how the nonspecific symptoms that often precede its diagnosis can lead to serious vascular events. At the same time, the presenting features in this case, such as young age, migraine, family history of early‐onset stroke, and multivessel cervical arteriopathy, illustrate that such a diagnosis need not remain hidden if an underlying vasculopathy is actively sought. In the absence of a known vasculopathy, anesthetic management required rapid decision‐making focused on cerebral perfusion, airway safety, and hemodynamic stability. The use of GA with manual in‐line stabilization, video laryngoscopy, and tightly controlled blood pressure proved appropriate in the setting of neurological deterioration and the need for urgent endovascular intervention. Retrospectively, the subsequent diagnosis of FMD reframes the intraoperative choices as particularly important and emphasizes the importance of maintaining a suspicion for vascular fragility in young stroke patients. For anesthesiologists, this case reinforces the importance of maintaining vigilance for occult vascular disease in young stroke patients and the necessity to have strategies prepared for such patients that minimize mechanical and hemodynamic stress.

## Funding

No funding was received for this study.

## Conflicts of Interest

The authors declare no conflicts of interest.

## Data Availability

The data that support the findings of this study are available on request from the corresponding author. The data are not publicly available due to privacy or ethical restrictions.

## Appendix A

**TABLE A1:** CARE checklist (2013).

Item	CARE topic	Reported?/location in manuscript
1	Title—the words “case report”	Yes ‐ Title
2	Key words	fibromuscular dysplasia; mechanical thrombectomy; anesthesia; cervical artery dissection; stroke
3	Abstract (background, case, conclusion)	Yes, Abstract
4	Introduction/background	Yes, Introduction
5	Patient information (demographics, history, symptoms)	Yes, Case History; augmented with young‐stroke history
6	Clinical findings	Yes, Case History; NIHSS/ASPECTS
7	Timeline	Yes, Table A2 (structured timeline)
8	Diagnostic assessment (methods, challenges, differential, prognosis)	Yes, Discussion
9	Therapeutic intervention (types, administration, changes)	Yes, Management
10	Follow‐up and outcomes (assessed outcomes, adherence, adverse events)	Yes, Outcomes and Follow‐up
11	Discussion (strengths/limitations, literature, rationale, takeaway)	Yes, Discussion
12	Patient perspective	Not obtained
13	Informed consent	Written informed consent was obtained from the patient for the publication of this case report and any accompanying images

**TABLE A2:** Structured patient timeline.

Time point	Event/findings/intervention
∼2 weeks before	Whiplash injury sustained at a concert
Night before presentation	Right‐sided headache
Day 0, presentation	Awoke with slurred speech and right‐sided facial numbness and extremity weakness; NIHSS 3
Day 0, initial imaging	CT brain + CTA head/neck: right ICA occlusion, high‐grade left ICA stenosis, left distal cervical vertebral artery dissection. ASPECTS: 10. Outside therapeutic window ⟶ admitted for medical/stroke care
Day 0, evening	Rapid response for new left‐sided weakness and facial droop; NIHSS 11; repeat CTA: increased attenuation in right MCA territory. Neurointerventional radiology consulted
Day 0/1, intervention	Emergent mechanical thrombectomy + stent under general anesthesia (in‐line stabilization, video laryngoscopy, arterial line). Peri‐procedural tirofiban; hemodynamics managed with esmolol, phenylephrine, ephedrine, glycopyrrolate
Postoperation	Extubated; transferred to SICU/neurocritical ICU.
Days 1–2	Neurocritical ICU monitoring
Day 3	Transferred to the regular nursing floor
Days 3–5	Brain‐to‐pelvis CTA: left proximal ICA dissecting pseudoaneurysm; left renal vein compression/left gonadal vein dilation
Day 6, discharge	Discharged on aspirin + clopidogrel × 3 months; dissection precautions; avoid air travel; referral to genetics and FMD clinic. mRS at discharge of 3
6 months postdischarge	Bilateral carotid artery ultrasound: new tortuous vessel at the distal right ICA and an aneurysm of 8 mm; comprehensive aortopathy genetic panel: no disease‐causing variants identified. FMD diagnosed
